# Leaf Morphological and Nutrient Traits of Common Woody Plants Change Along the Urban–Rural Gradient in Beijing, China

**DOI:** 10.3389/fpls.2021.682274

**Published:** 2021-08-26

**Authors:** Yuebo Su, Mark Renz, Bowen Cui, Xu Sun, Zhiyun Ouyang, Xiaoke Wang

**Affiliations:** ^1^State Key Laboratory of Urban and Regional Ecology, Research Center for Eco-Environmental Sciences, Chinese Academy of Sciences, Beijing, China; ^2^Shenzhen Academy of Environmental Sciences, Shenzhen, China; ^3^Department of Agronomy, University of Wisconsin-Madison, Madison, WI, United States; ^4^University of Chinese Academy of Sciences, Beijing, China; ^5^Beijing Urban Ecosystem Research Station, Chinese Academy of Sciences, Beijing, China

**Keywords:** urban plants, leaf functional traits, urban-rural gradient, soil nutrients, micro-climate

## Abstract

An increasing number of studies have found differences in the diversity of plant functional traits between urban and rural sites as a result of urbanization. However, the results remain inconsistent. In this study, we measured morphological and nutrient traits of 11 common woody plants along a continuous urban–rural gradient in Beijing, China. Leaf size (e.g., length, width, and area), specific leaf area, and leaf nitrogen and potassium contents decreased gradually and significantly along the urban–rural gradient, indicating that urbanization can enhance the capacity of plants to acquire resources for growth and production. Furthermore, soil nutrients and air temperature decreased along the urban–rural gradient, while air relative humidity increased. A structural equation model showed that these alterations in physical factors attributable to urbanization contributed directly or indirectly to changes in leaf functional traits, implying that changes in soil nutrients and micro-climate induced by urbanization may affect plant growth and production because of the improvement in resource acquisition capacity.

## Introduction

Global urbanization is accelerating rapidly with an unpredictable magnitude (Brenner and Keil, 2014), arousing public attention around the protection of urban ecosystems. Urban plants provide multiple ecosystem services for residents by mitigating heat stress and the occurrence of flooding, reducing air and water pollution, enhancing carbon sequestration and aesthetic value, and promoting human health (Clarke and Jenerette, 2015; Schwarz et al., 2017). In urban environments, plants suffer severe environmental stresses (e.g., heat, air pollution, and heavy metal stresses) (Polsky et al., 2014; Calfapietra et al., 2015; Zhang et al., 2019), but they also benefit from effective management practices (e.g., fertilization, irrigation, and pest control) (Gregg et al., 2003; Ziska et al., 2003; Zhao et al., 2016; Pretzsch et al., 2017). Understanding the way plants respond and adapt to urban environments is important not only for urban design and management but also for predicting plant responses to climate change (Farrell et al., 2015).

Changes in plant functional traits along an urban–rural gradient can help us understand the responses and adaptations of plants to urbanization (Perez-Harguindeguy et al., 2016; Cochard et al., 2019). Previous studies have focused primarily on comparing plant functional trait diversity between urban and rural sites (Searle et al., 2012; Alotaibi et al., 2020; El-Khatib et al., 2020), but consistency in results has not been achieved. Urbanization has been shown to induce significant increases in leaf area by 730% (Searle et al., 2012), specific leaf area by 47% (Song et al., 2019), and leaf nitrogen concentration by 15 to 23% (Nikula et al., 2010; Searle et al., 2011, 2012), and to cause no changes in leaf functional traits (Nikula et al., 2010; Searle et al., 2011; Leghari and Zaidi, 2013; Huang et al., 2016) or even decrease leaf area by 16 to 36% (van Rensburg et al., 1997; Pourkhabbaz et al., 2010; Leghari and Zaidi, 2013), specific leaf area by 12% (Lambrecht et al., 2016), and leaf nitrogen concentration by 18 and 33% (van Rensburg et al., 1997; Lambrecht et al., 2016). Plant responses to environmental gradients depend not only on the direction and extent of changes in environmental factors, but also on the biological characteristics of plants, such as their origin (Davidson et al., 2011; Díaz-Barradas et al., 2020) and life form (Lawrence, 2003; Ghimire et al., 2018). It is, therefore, imperative to investigate the direction and extent of changes in environmental factors along the urban–rural gradient (El-Khatib et al., 2020), and the origins and life forms of plant species of interest (Leghari and Zaidi, 2013). Previous studies have shown that non-native plants generally exhibit greater sensitivity to altered environmental conditions than native plants for achieving rapid establishment and spread in a new environment (Davidson et al., 2011; Díaz-Barradas et al., 2020). Plants with different life forms have different appearances, longevity, and growth rates, which influence their responses to environmental changes (Lawrence, 2003; Götmark et al., 2016; Ghimire et al., 2018).

Soil nutrients and micro-climate are the major environmental factors for explaining variations in leaf functional traits (Reich and Oleksyn, 2004; Wright et al., 2004, 2017; Song et al., 2019). For example, nutrient stress causes relatively small leaves to have a relatively low specific leaf area and leaf nutrient content (Cornelissen et al., 2003; Wright et al., 2004). Urbanization changes soil nutrients and micro-climate spatially. Monotonic increases in soil nutrients toward the city center have been reported in Beijing and Hubei, China (Li et al., 2013; Mao et al., 2014), and in New York, United States (Baxter et al., 2002), due to the input of construction waste and domestic waste, frequent irrigation with eutrophic reclaimed water, chemical fertilization, and invasion of exotic earthworms (Pouyat et al., 2010; Mao et al., 2013). The air temperature in urban areas is often 0.5 to 4°C higher than that in rural areas because of differences in biophysical features of the land and emissions of anthropogenic heat (Sakakibara and Owa, 2005; Yang et al., 2013). It is, therefore, necessary to investigate whether changes in soil nutrients and micro-climate along an urban–rural gradient affect plant trait variation.

In this study, we sampled 11 woody plant species from 13 public parks along a continuous urban–rural gradient in Beijing, China. We measured soil nutrients and continuously monitored micro-climatic factors in each park. The objectives of this study were to examine changes in leaf morphological and nutrient traits along the urban–rural gradient, and to determine whether these changes depend on the life form and origin of species, and are related to local soil nutrients and micro-climate.

## Methods

### Study Area and Sites

**Figure 1:**
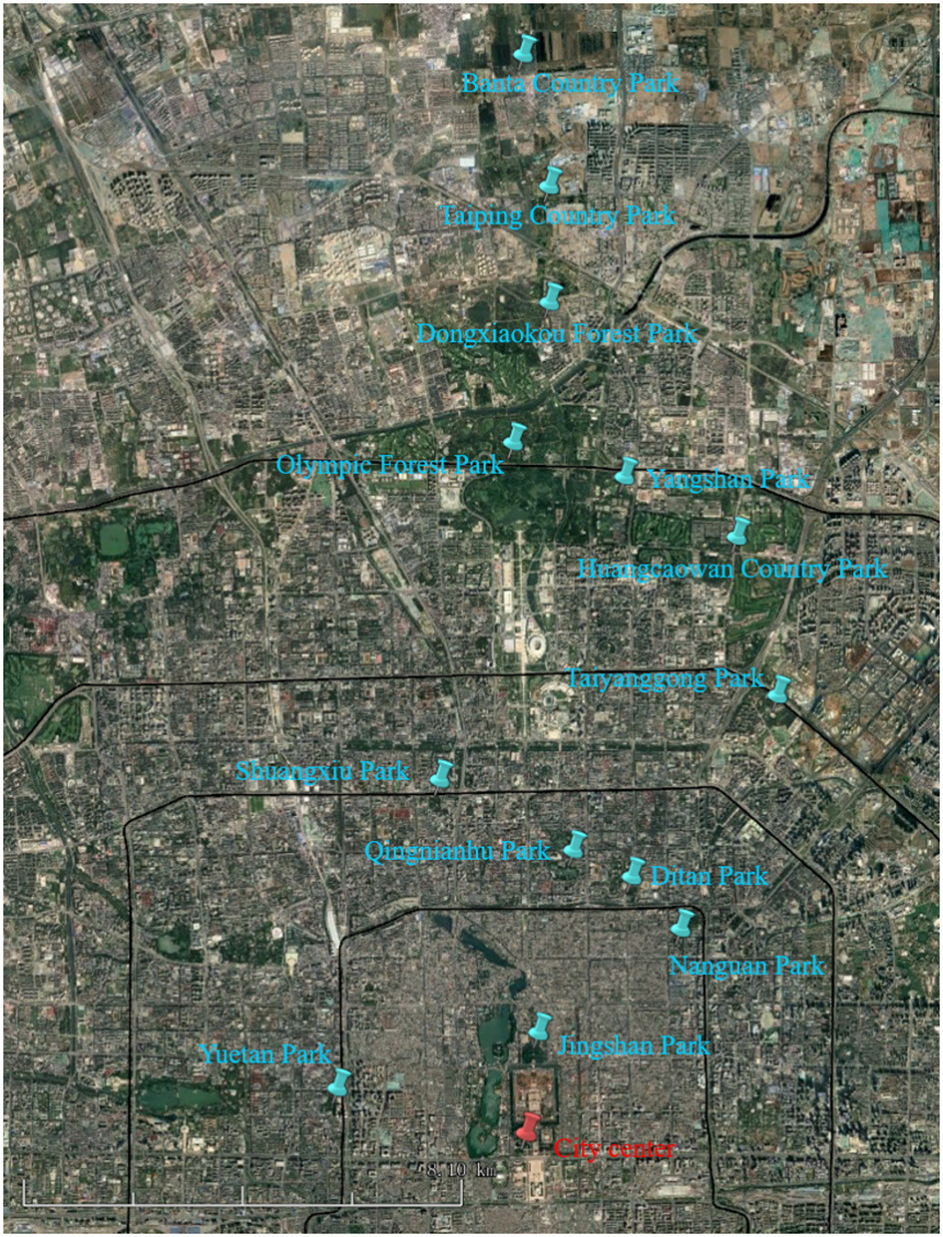
Location of the 13 public parks selected (Background image from Google Earth).

This study was conducted in the northern part of Beijing, the capital of China (40°00′N, 116°20′E), which has a typical temperate, semi-humid, continental monsoon climate. The average annual precipitation and temperature are ~550 mm and 12°C, respectively (The National Meteorological Information Center of China Meteorological Administration). Public parks are an important land-use type in cities, hosting substantial levels of plant diversity and playing essential roles in providing recreational services for local residents. In Beijing, there are more than 200 public parks, widely located in urban and suburban regions, which provide a great opportunity for studying the responses of plant traits to urbanization. Thirteen public parks were selected randomly along a south–north axis of urban development representing di?erent urbanization intensities (Figure 1 and Supplementary Table 1; Peng et al., 2016). These parks were between 2.7 and 20.3 km from the city center. The following aspects were considered in choosing the public parks for investigation: (1) the parks were located in the transect of the urban–rural gradient with significant differences in urbanization density; (2) the parks were spaced at least 1 km apart to avoid spatial autocorrelation; and (3) the parks were allowed to install micro-climate measurement instruments and collect soil and plant samples.

### Selection of Species and Measurement of Traits

Eleven woody plant species were selected, comprising native trees (*Salix babylonica* L., *Sophora japonica* L., *Acer truncatum* Bunge, *Pinus tabuliformis* Carr., and *Fraxinus chinensis* Roxb.), native shrubs [*Lonicera maackii (*Rupr.) Maxim., *Forsythia suspensa* (Thunb.) Vahl, and *Lagerstroemia indica* L.], non-native trees (*Ginkgo biloba* L.), and non-native shrubs [*Kerria japonica* (L.) DC. and *Jasminum nudiflorum* Lindl.]. The following factors were considered in the selection of woody plant species. (1) Dominance: the species represent the dominant woody plant species in urban forests. (2) Distribution: the species occur in almost all the parks investigated along the urban–rural gradient. (3) Functional type: the species have different origins (e.g., non-native and native species) and life forms (e.g., tree and shrub).

Eight leaf functional traits were chosen, leaf length, width, and area; specific leaf area (SLA); and leaf carbon (leaf C), leaf nitrogen (leaf N), leaf phosphorus (leaf P), and leaf potassium (leaf K). These leaf functional traits are the most common and essential characteristics of plants and play important physiological roles in plant growth and production. These traits are also sensitive to environmental changes, especially urbanization. Previous studies have revealed significant differences in leaf size (van Rensburg et al., 1997; Pourkhabbaz et al., 2010; Searle et al., 2012; Leghari and Zaidi, 2013), in SLA (Lambrecht et al., 2016; Song et al., 2019), and in leaf N and P concentrations (Nikula et al., 2010; Searle et al., 2011, 2012; Huang et al., 2016; Lambrecht et al., 2016) between urban and rural sites.

In early September 2018, three mature and healthy individuals of each species were selected randomly from each site for the measurement of leaf functional traits. The selected individuals within a species had similar features, such as height and diameter at breast height (Supplementary Table 2). Tree health was determined according to the method of Boa (2003), and 100 to 200 fully expanded and sun-exposed leaves were collected from each individual (Cornelissen et al., 2003).

Ten leaves were chosen randomly from each individual to measure leaf length, width, and area, and SLA. Leaf length, width, and area were measured using a LI-3000C area meter (LI-COR, Lincoln, NE, United States). The dry weight of leaves was measured after oven-drying for 72 h at 65°C. SLA was calculated as the leaf area divided by its dry weight. The other leaves collected were air-dried and ground to analyze leaf C, N, P, and K contents. Leaf C and N contents were determined using an automatic elemental analyzer (Vario EL III; Elementar, Langenselbold, Germany), while leaf P and K contents were measured by inductively coupled plasma optical emission spectroscopy (ICP-OES) using Prodigy (Leeman, Mason, OH, United States) after digestion with freshly distilled concentrated HNO_3_/H_2_O_2_ in a microwave oven (Oliva et al., 2003; Merilä and Derome, 2008).

### Measurement of Environmental Factors

Air temperature and relative humidity were measured in each park at 10-min intervals using a mini data-logger (HOBO U23 Pro v2 Temperature/Relative Humidity Data Logger-U23-001, Onset Computer Corporation, Bourne, MA, United States). Daily mean air temperature and relative humidity were recorded from June 1 to August 31, 2018, as this is the period with highest probability of summer heat that can be detrimental to plant functional traits (Duan et al., 2014).

Four soil nutrient variables were measured: soil organic C, total N, and available P and K contents. Surface soil (0–20 cm) samples from three locations around each individual plant were combined to yield one composite soil sample in early September 2018. The soil samples were oven-dried to constant weight at 105°C for 72 h and sieved. Soil organic C content was measured using the dilution heat K_2_Cr_2_O_7_ oxidation volumetric method (Lu, 2000), and soil N content was measured using the semi-micro-Kjeldahl method (Lu, 2000). Soil was digested with 0.5 mol/l pH 8.5 NaHCO_3_ and available P content was analyzed by molybdenum-antimony anti-colorimetry (Lu, 2000). Soil was digested with 1 mol/l pH 7 CH_3_COONH_4_. Available K content was determined by ICP-OES using Prodigy (Leeman, Mason, OH, United States) (Lu, 2000).

### Data Analysis

To explore changes in leaf traits along the urban–rural gradient, a generalized linear mixed model was fitted with trait as the response variable, distance from the city center as the fixed effect, and species as the random effect (gamma distribution with LOG LINK function). Likelihood ratio tests of a full model against a null model were performed to measure the significance of the fixed effect (Fajardo and Siefert, 2018). The explanatory power of the models was evaluated by calculating the marginal *R*^2^ and conditional *R*^2^ (Nakagawa and Schielzeth, 2013).

Changes in leaf traits along the urban–rural gradient for each species were determined by fitting general linear models, with traits as the response variables and distance as the predictor variable. Traits showing significant and insignificant changes along the urban–rural gradient for non-native species, native species, shrubs, and trees were counted, and two 2 × 2 contingency tables were constructed, one with the origin of species (non-native or native) as the rows and changes along the urban–rural gradient as the columns, and the other with the life form of species (shrubs or trees) as the rows and changes along the urban–rural gradient as the columns. Fisher's exact test was performed for the 2 × 2 contingency tables to test whether the sensitivity of traits to the urban–rural gradient was different between non-native and native species, and between shrubs and trees.

General linear models were fitted with climate and soil factors as the response variables and distance from the city center as the predictor variable to analyze changes in climate and soil factors along the urban–rural gradient. Structural equation modeling (SEM) was then performed to assess direct and indirect effects of soil nutrients and micro-climate on leaf functional traits. It is anticipated that the intensity of urbanization (distance from the city center) affects soil nutrients and micro-climate, which in turn affect leaf functional traits (Reich and Oleksyn, 2004; Wright et al., 2004, 2017; Song et al., 2019). It is also hypothesized that changes in leaf morphological traits are induced by changes in leaf nutrient traits (Tian et al., 2016; Rota et al., 2018). Leaf length, width, and area, and SLA were incorporated into a latent variable, morphological traits, reflecting structural and physical characteristics of leaves. Leaf C, N, P, and K concentrations were incorporated into another latent variable, nutrient traits, representing the concentration of nutrient elements in leaves. Although SLA was closely related to leaf nutrient contents (Supplementary Figure 1), SLA was still incorporated into the morphological traits variable, because SLA often determines the physical structure of leaves together with leaf size (Cornelissen et al., 2003; Griffin-Nolan et al., 2018). A prior model based on a known theoretical construct is shown in Supplementary Figure 2. Different individuals in a park shared the same micro-climate data. We adopted several indices to evaluate model suitability: chi-squared/degree of freedom ratio (CMIN/DF ≤ 3), goodness of fit index (GFI ≥ 0.9), comparative fit index (CFI ≥ 0.9), root mean squared error of approximation (RMSEA ≤ 0.1), and Akaike information criterion (AIC; Grace, 2006; Ji and Zhang, 2009). Associations between non-significant parameters were removed to determine the best-fit models. Including soil nutrients and micro-climate in one model produced a model with poor suitability (CMIN/DF = 4.039, GFI = 0.793, CFI = 0.897, RMSEA = 0.152); these effects were therefore analyzed separately. SEM analyses were performed using the AMOS v. 22 software. Other analyses were performed using R (R, v. 3.5, http://www.R-project.org).

## Results

### Changes in Leaf Morphological and Nutrient Traits Along the Urban–Rural Gradient

Leaf length, width, and area, SLA, and leaf N and K contents decreased along the urban–rural gradient (*p* < 0.001), while leaf P content increased (*p* < 0.001, Table 1 and Supplementary Figure 3).

**Table 1:** Changes in leaf functional traits along the urban–rural gradient across all the species studied, which was fitted with generalized linear mixed models, with traits as the response variable, distance of sites from the city center as the fixed effect, and species as the random intercept.

**Traits**	**Slope of changes**	**Marginal *R^**2**^***	**Conditional *R^**2**^***	**Chisq**	***P*-value**
Length	−0.006	0.02	0.37	16.44	** <0.001**
Width	−0.009	0.02	0.63	30.48	** <0.001**
Area	−0.018	0.04	0.51	39.23	** <0.001**
SLA	−0.015	0.05	0.39	41.75	** <0.001**
Leaf C	0.000	0.00	0.29	1.12	0.29
Leaf N	−0.010	0.07	0.27	42.49	** <0.001**
Leaf P	0.012	0.03	0.20	15.58	** <0.001**
Leaf K	−0.035	0.19	0.34	101.10	** <0.001**

*Note: The bold p-value indicated that the change of traits along the urban-rural gradient was significant*.

**Figure 2:**
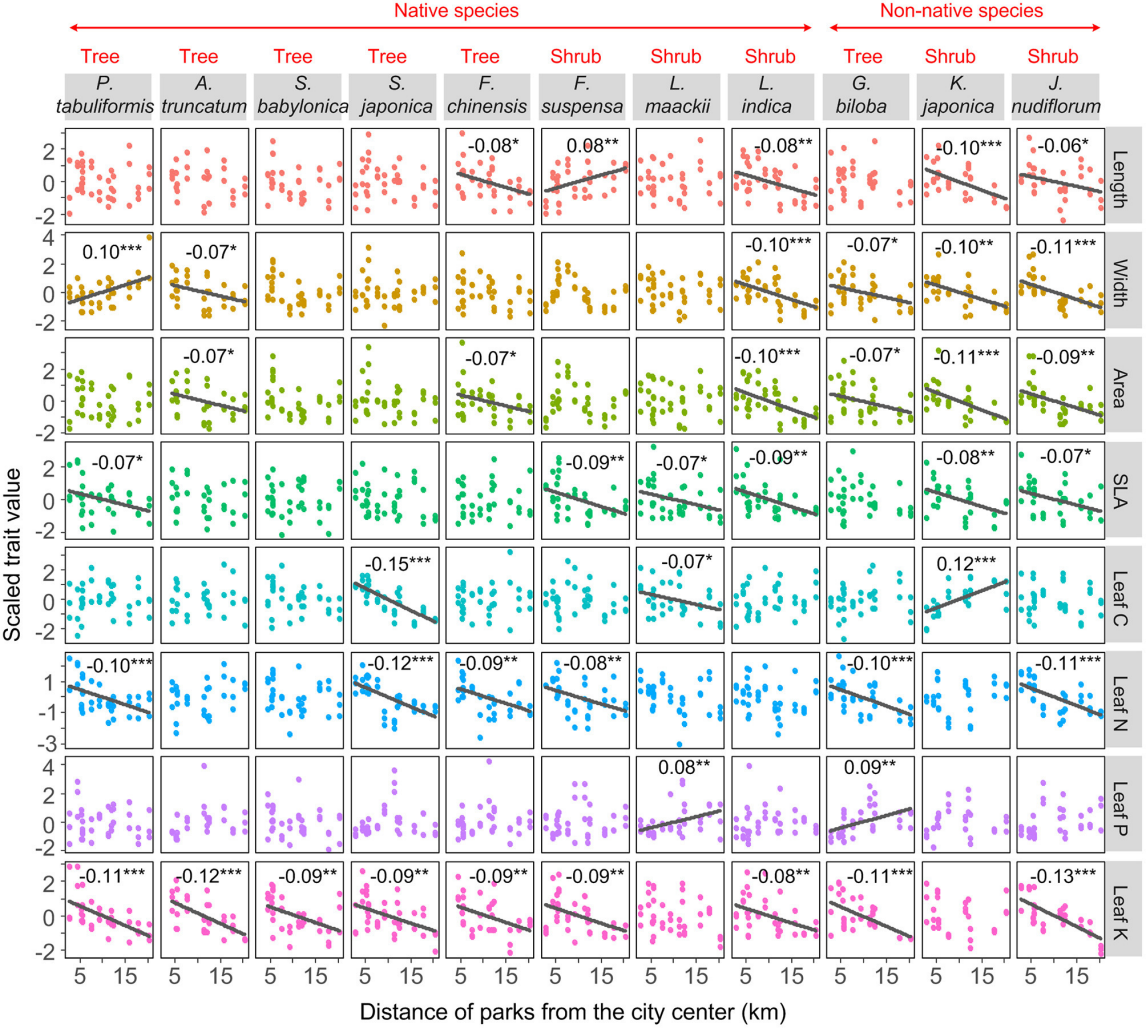
Changes in leaf functional traits along the urban–rural gradient for each species, fitted with general linear models, with trait as the response variable and distance as the predictor variable. Trait values were scaled to zero mean and unit standard deviation in order to plot them on the same scale. The sign is the slope of changes in leaf functional traits along an urban–rural gradient. Regression lines and slopes are shown when changes are significant (***p < 0.001, **p < 0.01, *p < 0.05).

The urban–rural gradient of leaf functional traits varied across species and traits (Figure 2). Leaf morphological traits of non-native species and shrubs were more sensitive to the urban–rural gradient than those of the native species (Fisher's exact test, OR = 6.98, *p* = 0.017) and trees (Fisher's exact test, OR = 5.73, *p* = 0.008). There was no difference in the sensitivity of leaf nutrient traits to the urban–rural gradient between the non-native species and the native species (Fisher's exact test, OR = 1.28, *p* = 0.746) or between trees and shrubs (Fisher's exact test, OR = 0.67, *p* = 0.556). Leaf K content showed the greatest sensitivity to the gradient (nine species showed urban–rural changes in leaf K content), followed by leaf width and area, SLA, and leaf N content (six species exhibited changes in these traits; Figure 2).

### Changes in Soil Nutrients and Micro-Climate Along the Urban–Rural Gradient and Their Effects on Leaf Morphological and Nutrient Traits

**Figure 3:**
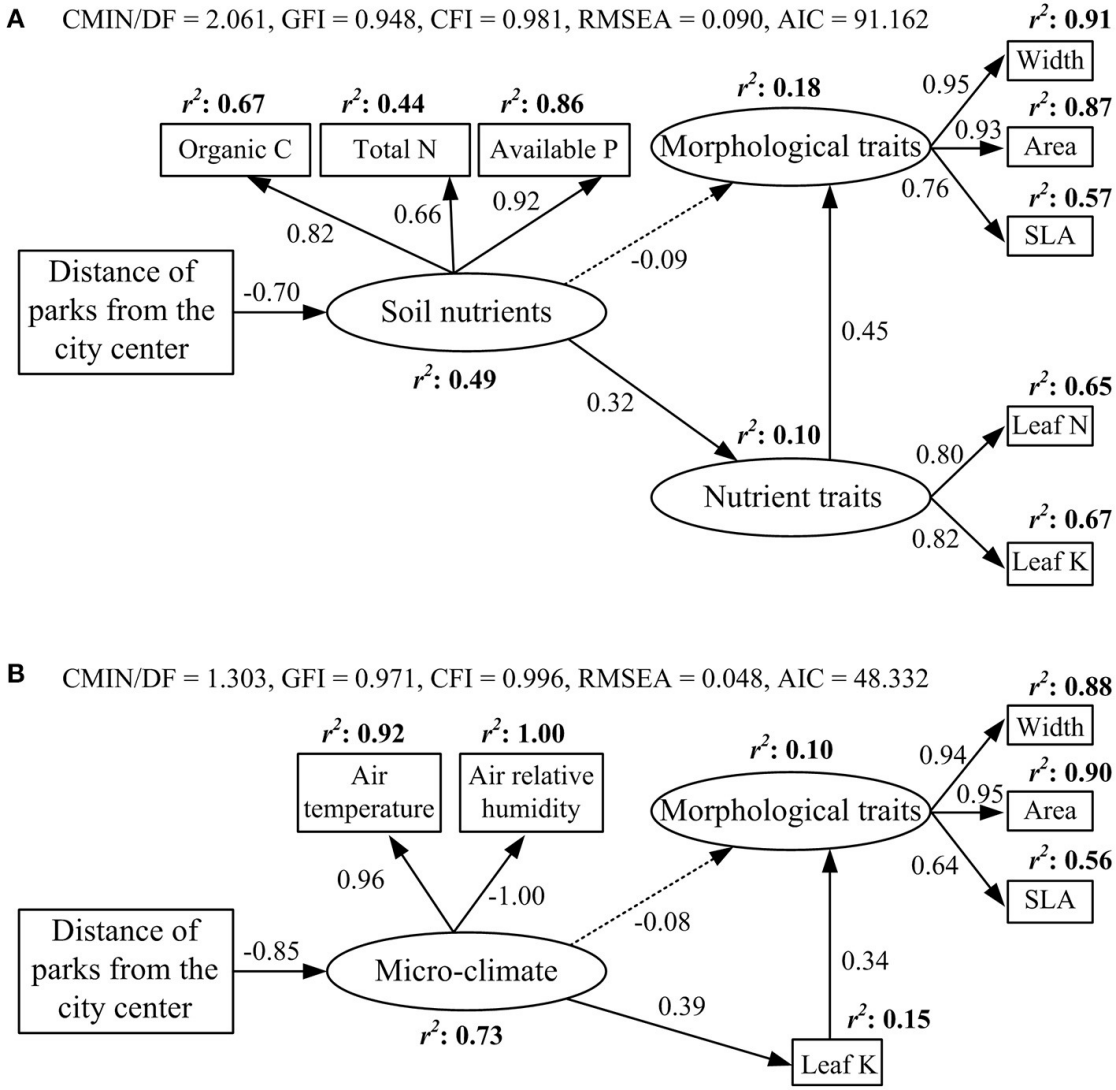
Effects of **(A)** soil nutrients and **(B)** micro-climate on leaf morphological and nutrient traits based on the structural equation model. Solid and dashed arrows indicate significant and insignificant effects, respectively (*p* < 0.05). Values on arrows indicate standardized regression weights.

Soil nutrient contents (soil organic carbon, total nitrogen, and available phosphorus and potassium) and air temperature decreased along the urban–rural gradient, but air relative humidity increased (*p* < 0.01, Supplementary Figure 4). The final SEM model for soil nutrients and functional traits explained 10% of the variation in nutrient traits and 18% of the variation in morphological traits (Figure 3A). Distance from the city center had a direct negative effect on soil nutrients (standardized regression weight: −0.7). Soil nutrients had a direct positive effect on nutrient traits (0.32) and an indirect positive effect on morphological traits *via* nutrient traits (0.14). Nutrient traits had the strongest direct effect on morphological traits (0.45). The final SEM model for micro-climate and functional traits accounted for 15% of the variation in leaf K content and 10% of the variation in morphological traits (Figure 3B). Distance from the city center had a direct negative effect on micro-climate (−0.85). Micro-climate had a direct positive effect on leaf K content (0.39) and an indirect positive effect on morphological traits *via* leaf K content (0.13). Leaf K content had the strongest effect on morphological traits (0.34).

## Discussion

### Leaf Morphological and Nutrient Traits Change Along the Urban–Rural Gradient

Urbanization influences not only species diversity of plants (Aronson et al., 2014), but also trait variation. Decreases in leaf size (length, width, and area), SLA, and leaf N and K contents along the urban–rural gradient imply that plants growing in urban parks tend to have an acquisitive resource-use strategy (Wright et al., 2004) to enhance their competition, growth, and production (Searle et al., 2012; Wright et al., 2017), subsequently influencing ecosystem function. The decrease in leaf area identified along the urban–rural gradient in Beijing is consistent with findings for *Quercus rubra* seedlings (Searle et al., 2012), but in contrast to results for *Platanus orientalis* (Pourkhabbaz et al., 2010). SLA decreased along the gradient, similar to observations for subtropical monsoon evergreen broad-leaved forests in Guangzhou, China (Song et al., 2019). Decreases in leaf N content followed the same patterns found in previous studies showing that *Populus tremula* (Nikula et al., 2010), *Q. rubra* (Searle et al., 2012), and *Crepis sancta* (Lambrecht et al., 2016) have higher leaf N content in urban areas than their counterparts in rural locations. However, changes in SLA of European aspen (*P. tremula*) (Nikula et al., 2010) and 18 French urban plant species (Cochard et al., 2019) were not seen along an urban–rural gradient, possibly owing to differences in plant species and the urban–rural gradient.

Leaf phosphorus content increased gradually along the urban–rural gradient. P is a major component of nucleic acids, sugar phosphates, adenosine triphosphate, and phospholipids, all of which play important roles in photosynthesis, which is related closely to rapid growth (Bieleski, 1973; Reich et al., 2009). Lack of P can restrict the relationship between photosynthetic capacity and N by limiting ribulose-1,5-bisphosphate regeneration (Reich et al., 2009). Lower leaf P content in urban parks compared with rural parks might counteract plant growth enhanced by greater leaf size and N and K contents in urban parks.

### Plant Sensitivities to the Urban–Rural Gradient Vary Across Species and Traits

We found that leaf morphological traits of non-native species were more sensitive to the urban–rural gradient than those of native species (Figure 2). Similarly, non-native *Oenothera drummondii* populations show greater sensitivity to water stress (leaf relative water content decreased to 80%) than populations of native species (70%) (Díaz-Barradas et al., 2020). The greater sensitivity of non-native species to environmental gradients corresponds to their large trait variations compared with native species (Funk, 2008; Davidson et al., 2011). Such large variations allow non-native species to acclimate to various environmental conditions, cope better in a novel environment, and outcompete native species (Funk, 2008; Davidson et al., 2011).

Leaf morphological traits of shrubs were more sensitive to the urban–rural gradient than those of trees. This might be because (1) trait plasticity is higher for shrubs than for trees (Götmark et al., 2016; Ghimire et al., 2018), (2) the management intensity of shrubs is often more intensive than that of trees for esthetic reasons, and (3) shrubs may be more sensitive to environmental stresses than trees, e.g., greater sensitivities to herbivore damage and pollutants due to a relatively short life cycle (Laurence et al., 1994; Maron and Crone, 2006). Similarly, Ghimire et al. (2018) found that shrubs exhibited a much stronger response to declining soil water contents than trees did, because shrubs have shallower roots.

Sensitivity to the urban–rural gradient varied across traits. Few studies have evaluated the response of leaf K content to environmental changes (Wright et al., 2004; Song et al., 2019), despite K, the most abundant cation in plant tissues, playing important roles in plant growth, such as enhancing cell membrane stability, reducing leaf water loss, and improving phloem transport of carbohydrates and photosynthesis (Steven, 1985; Wu et al., 2019). Among all the traits studied here, leaf K content exhibited the greatest sensitivity to the urban–rural gradient (Table 1 and Figure 2). This implies that the capacity of plants to regulate leaf K content may be greater or more easily regulated than that of the other traits. Environmental changes along the urban–rural gradient may also have a stronger effect on leaf K content than on other traits. However, no response of leaf C content was observed (*p* = 0.29, Table 1). This might be expected, because leaf C content, a trait that provides plant structure, often exhibits greater stability than other traits that commonly limit plant growth (e.g., leaf N content; (Knecht and Göransson, 2004; Ågren, 2008; Derroire et al., 2018).

### Soil Nutrients and Micro-Climate Influence Leaf Morphological and Nutrient Traits

Soil nutrients (soil organic carbon, total nitrogen, and available phosphorus and potassium) decreased along the urban–rural gradient. Major reasons for this might be that (1) inputs of atmospheric deposition and domestic waste result in increased soil nutrients in urban areas (Phoenix et al., 2012; Mao et al., 2014), and (2) accelerated soil nutrient cycling due to invasion of exotic earthworms and high soil temperatures caused by the heat island effect facilitate the accumulation of soil nutrients in urban areas (Baxter et al., 2002; Pouyat et al., 2002, 2010; Li et al., 2013; Phillips et al., 2019). Other reasons may be element-specific. Older soils in urban parks may accumulate more soil organic C than younger soils in rural parks (Mao et al., 2014). Construction materials (i.e., bricks and cement) with high Ca may also indirectly increase soil available K in urban parks, because divalent Ca has higher surface charge densities and cation exchange interactions than monovalent K, allowing K ions to be freed in soil (Moussa et al., 2009; Mao et al., 2014).

Urbanization changes multiple biotic and abiotic environmental factors over short distances (Pouyat et al., 2010; Aronson et al., 2014; Wang et al., 2019). Changes in any of these factors may lead to changes in selection pressures exerted on plant traits and resource-use strategies. We found that increases in soil nutrient contents and air temperature, and decreases in air relative humidity attributable to urbanization contributed directly or indirectly to changes in plant traits closely related to the improved resource acquisition capacity of urban plants (greater leaf length, area, SLA, and leaf N and K contents) (Table 1 and Figures 2, 3) (Wright et al., 2004). This is consistent with previous studies that found urbanization favors plant species and individuals with the ability to quickly acquire resources related to higher soil fertility (Song et al., 2019) and higher air temperature (Zhu et al., 2020) in urban areas.

Soil nutrients have a direct effect on leaf nutrient traits, but not morphological traits (Figure 3A). This is consistent with the results of Firn et al. (2019), who found that nutrient addition treatments could explain considerable amounts of variation in leaf nutrient contents, but not in SLA. This confirms that plants may prioritize the regulation of leaf nutrient traits to adapt to soil nutrient variability, because leaf nutrient regulation is often easier and requires less time than leaf morphological regulation (Grime and Mackey, 2002).

We found that micro-climate had a strong direct influence on leaf potassium content, but not on the other nutrient traits (Figure 3B). Hotter and drier air conditions, in part, promote an increase in leaf K content in urban areas. This increase helps plants overcome unfavorable environments (e.g., drought), because higher leaf K content is beneficial, protecting osmosis and controlling leaf water loss through stomatal regulation (Wu et al., 2019). Micro-climate had an indirect positive effect on morphological traits *via* change in leaf K content. A possible explanation for this is that increased temperature was conducive to increasing the activity of plant metabolic enzymes, photosynthetic metabolism, and leaf nutrient contents, resulting in an increase in leaf length and area, and SLA (Wright et al., 2017).

Leaf morphological traits were indirectly influenced by environmental factors (soil nutrients and micro-climate; Figure 3) because there were strong associations between morphological traits and nutrient traits (Supplementary Figure 1). Niinemets and Kull (2003) also identified relationships between leaf structure and nutrients in temperate shrubs and trees, with leaf K content associated strongly with morphological traits (SLA) on a local scale.

Leaf carbon and phosphorus contents were not included in the final structural equation models (Figure 3). We observed no significant changes in leaf C content along the urban–rural gradient. Leaf C content is an inherently conserved character found to be stable across plant taxa (Knecht and Göransson, 2004) and development stages (Derroire et al., 2018). Along the urban–rural gradient, leaf P content increased significantly, but soil available P content decreased significantly. This suggests that soil nutrients might not be the major factors influencing leaf P content. Luo et al. (2016) also found no evidence that intraspecific variation in leaf P content was correlated with soil resources or climatic conditions in 55 tree species in subalpine forests in Yulong Mountain, China. The urban–rural gradient of leaf P content, therefore, requires further investigation. From the results of SEMs (Figure 3), soil nutrients and micro-climate influenced leaf nutrient traits significantly but leaf morphological traits insignificantly, and the standardized regression weights between soil nutrients and leaf nutrient traits, and between micro-climate and leaf nutrient traits were 0.32 and 0.39, respectively, indicating that micro-climate had a greater effect on leaf traits than on soil nutrients.

Because leaf size, SLA, and leaf N and K contents are positively related to resource acquisition of plants (e.g., light, CO_2_, and nutrients), the enhancement of these leaf functional traits in urban parks could be of benefit to plant growth, and subsequently increase urban ecosystem services, such as carbon sequestration, climatic and hydrological regulations, and aesthetic value (Cornelissen et al., 2003; de Bello et al., 2010). However, the accelerated growth of urban plants would pose some risks and challenges for urban vegetation managements. Plants that grow fast would consume more water and be more sensitive to damages from herbivore and bad weather (Laurence et al., 1994; Maron and Crone, 2006; Pretzsch et al., 2017). In urban environments with dry and hot conditions, plants would be more likely to be threatened by drought. Hence, more management measures should be taken, such as irrigation and pesticide application. Plants that grow fast also need more pruning to prevent the impacts of plant canopy on transportation and landscape. Because leaf morphological traits of non-native species are more sensitive to the urban–rural gradient than those of native species, non-native species should not be recommended when selecting plant species in urban greening.

Although the results are limited by the investigation of only 11 species sampled from 13 parks in Beijing, they clearly show general patterns of variation in micro-climate, soil nutrient, leaf size, and nutrient contents along the urban–rural gradient. Because changes in leaf functional traits along an urban–rural gradient vary with species, trait, and urban context, it is impossible to arrive at an identical spatial pattern suitable globally for depicting all changes in plant functional traits along any urban–rural gradient. More field studies using different plants from different cities are required. A meta-analysis will be crucial for figuring out what we know currently and identifying differences among the studies available.

## Conclusions

Leaf size (length, width, and area), SLA, and leaf N and K contents decreased gradually along a continuous urban–rural gradient, implying that urban plants tend to have an acquisitive resource-use strategy. Leaf traits of non-native species were more sensitive to the urban–rural gradient than those of native species. Because leaf K content was most sensitive to the gradient, this can be used as a good indicator for monitoring plant responses to urbanization. Alteration in soil nutrients and micro-climatic conditions attributable to urbanization affected changes in leaf traits directly or indirectly. The findings reveal that plants can adjust their functional traits to acquire resources and adapt to urban environments quickly, thus, increasing their growth and production.

## Data Availability Statement

The original contributions presented in the study are included in the article/Supplementary Material, further inquiries can be directed to the corresponding author.

## Author Contributions

YS, ZO, and XW designed the study. YS, BC, and XS performed the experiment and analyzed the data. YS, MR, and XW prepared and revised the manuscript.

## Conflict of Interest

The authors declare that the research was conducted in the absence of any commercial or financial relationships that could be construed as a potential conflict of interest.

## Publisher's Note

All claims expressed in this article are solely those of the authors and do not necessarily represent those of their affiliated organizations, or those of the publisher, the editors and the reviewers. Any product that may be evaluated in this article, or claim that may be made by its manufacturer, is not guaranteed or endorsed by the publisher.

## Supplementary Material

Supplementary Figure 1 Pearson correlation matrix of the leaf functional traits. Trait values were scaled to zero mean and unit standard deviation in order to plot them on the same scale. (^***^*p* < 0.001, ^**^*p* < 0.01, ^*^*p* < 0.05).

Supplementary Figure 2 Hypothetical model of the structural equation model for effects of **(A)** soil nutrients and **(B)** micro-climate on leaf morphological and nutrient traits. Variables in oval and rectangular boxes are latent and observed variables, respectively. Single-headed arrows indicate a causal influence of one variable on another.

Supplementary Figure 3 Changes in leaf functional traits along the urban–rural gradient for all the selected species. Trait values were scaled to zero mean and unit standard deviation in order to plot them on the same scale. Solid and dashed arrows indicate significant and insignificant effects, respectively (*p* < 0.05).

Supplementary Figure 4 Changes in **(A–D)** soil nutrients and **(E,F)** micro-climate along the urban–rural gradient, fitted with general linear models, with environmental factor as the response variable and distance as the predictor variable.

Supplementary Table 1 Basic profiles of the public parks selected.

Supplementary Table 2 Plant height and diameter at breast height.
