# Supplementary figures and images for: Leaf Morphological and Nutrient Traits of Common Woody Plants Change Along the Urban–Rural Gradient in Beijing, China
Source: Front Plant Sci. 2021 Aug 26;12:682274. doi: 10.3389/fpls.2021.682274 (PMC8427184; doi:10.3389/fpls.2021.682274)

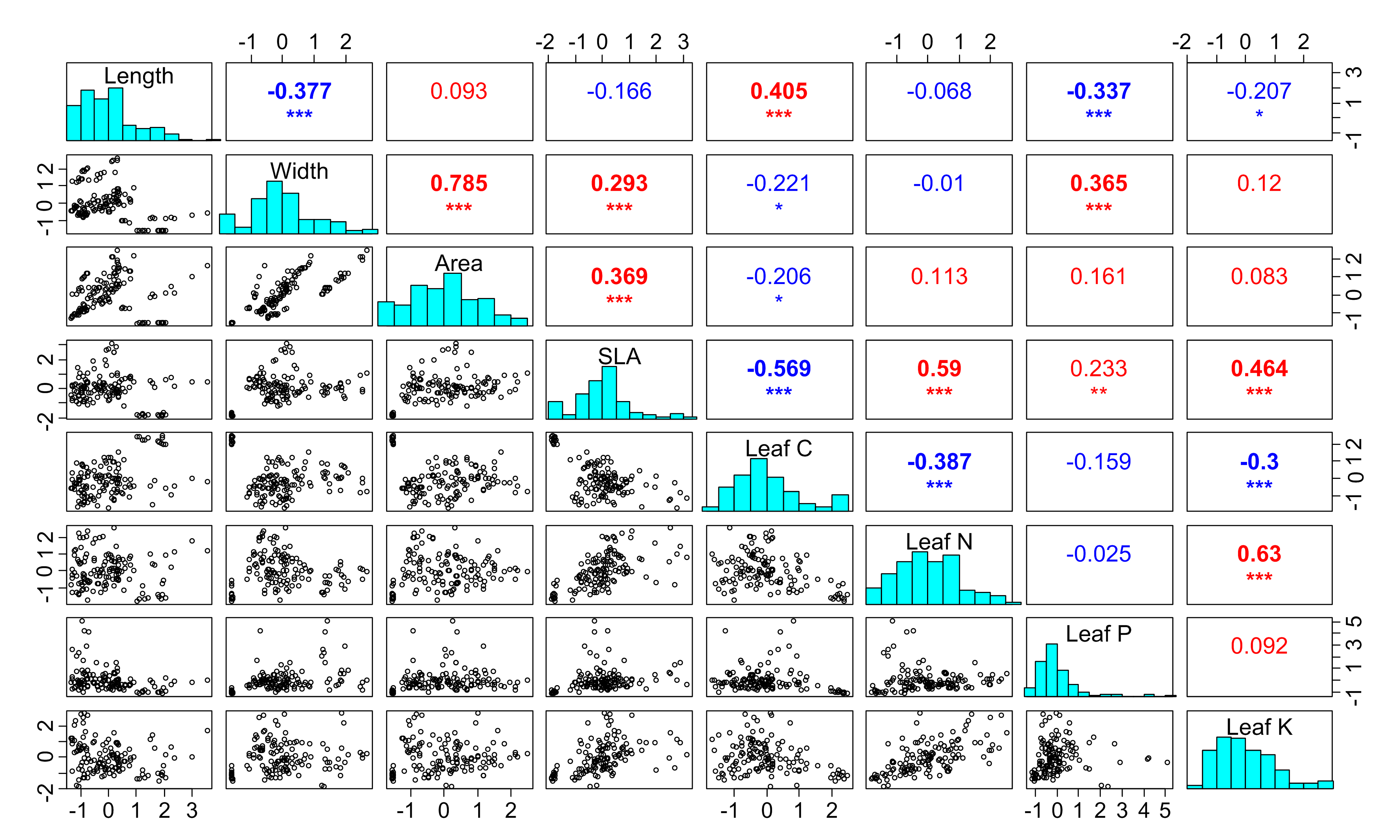

Supplement: Supplementary Figure 1 — Pearson correlation matrix of the leaf functional traits. Trait values were scaled to zero mean and unit standard deviation in order to plot them on the same scale. (***p < 0.001, **p < 0.01, *p < 0.05). [file Image_1.TIF]

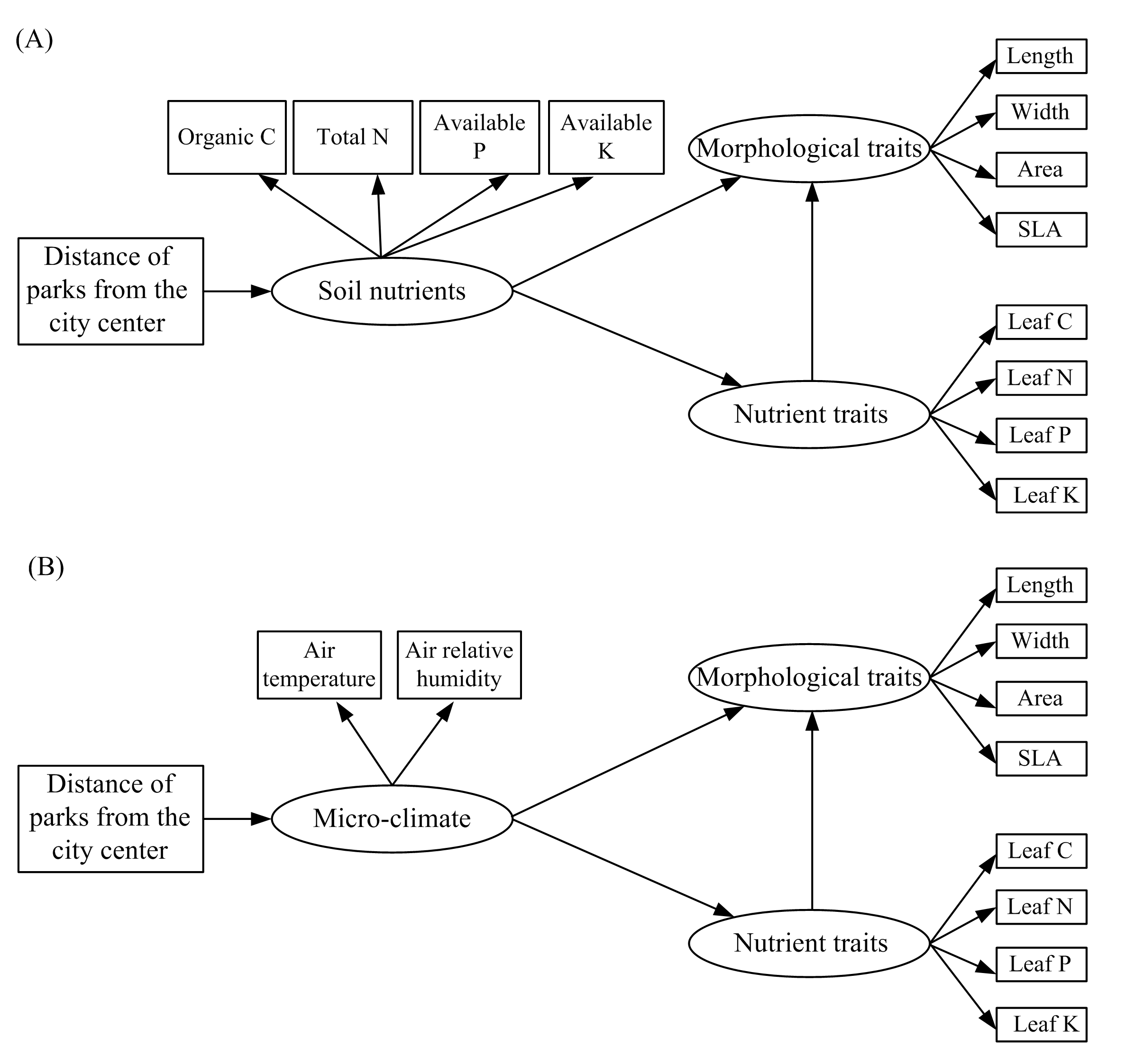

Supplement: Supplementary Figure 2 — Hypothetical model of the structural equation model for effects of (A) soil nutrients and (B) micro-climate on leaf morphological and nutrient traits. Variables in oval and rectangular boxes are latent and observed variables, respectively. Single-headed arrows indicate a causal influence of one variable on another. [file Image_2.TIF]

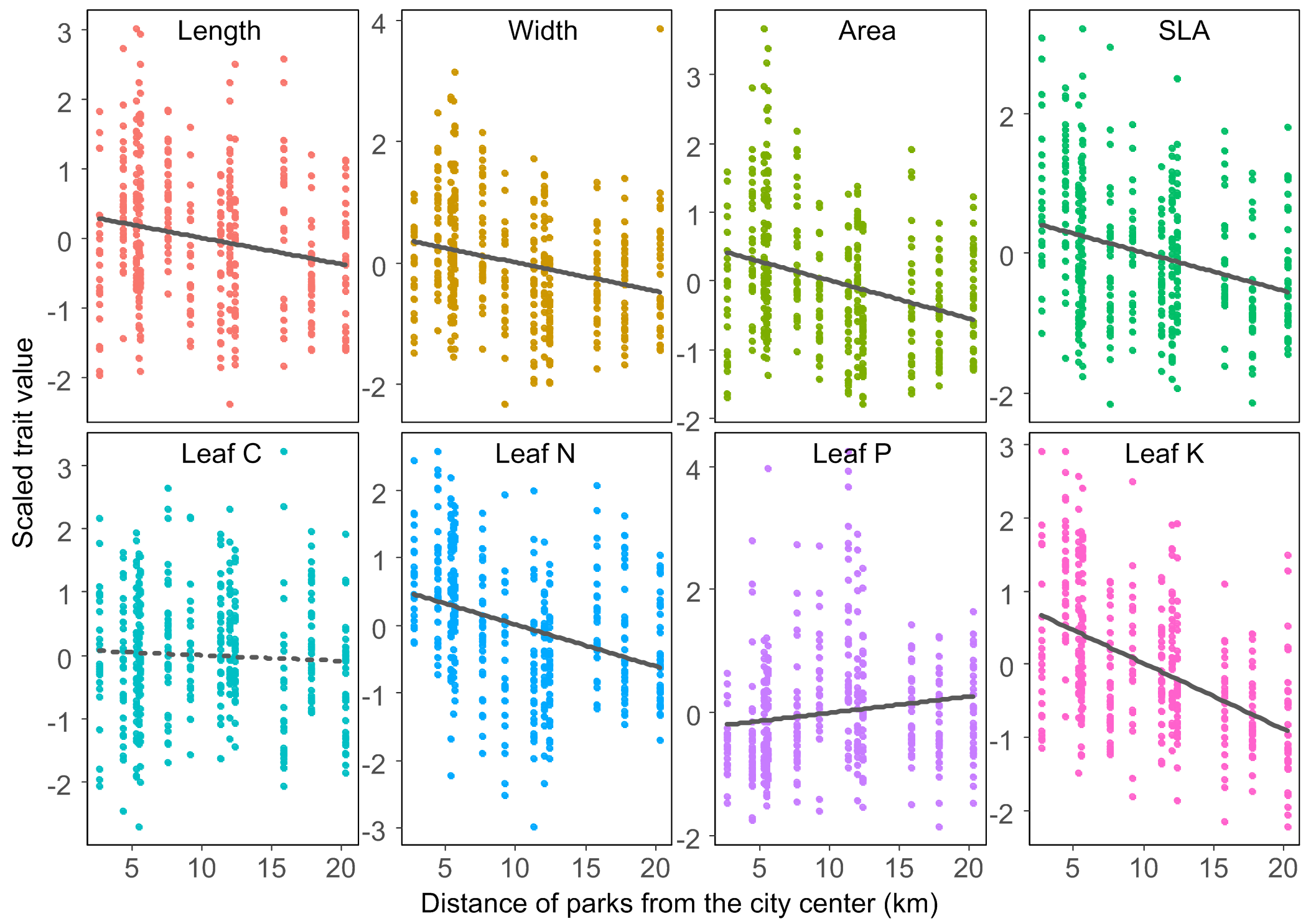

Supplement: Supplementary Figure 3 — Changes in leaf functional traits along the urban–rural gradient for all the selected species. Trait values were scaled to zero mean and unit standard deviation in order to plot them on the same scale. Solid and dashed arrows indicate significant and insignificant effects, respectively (p < 0.05). [file Image_3.TIF]

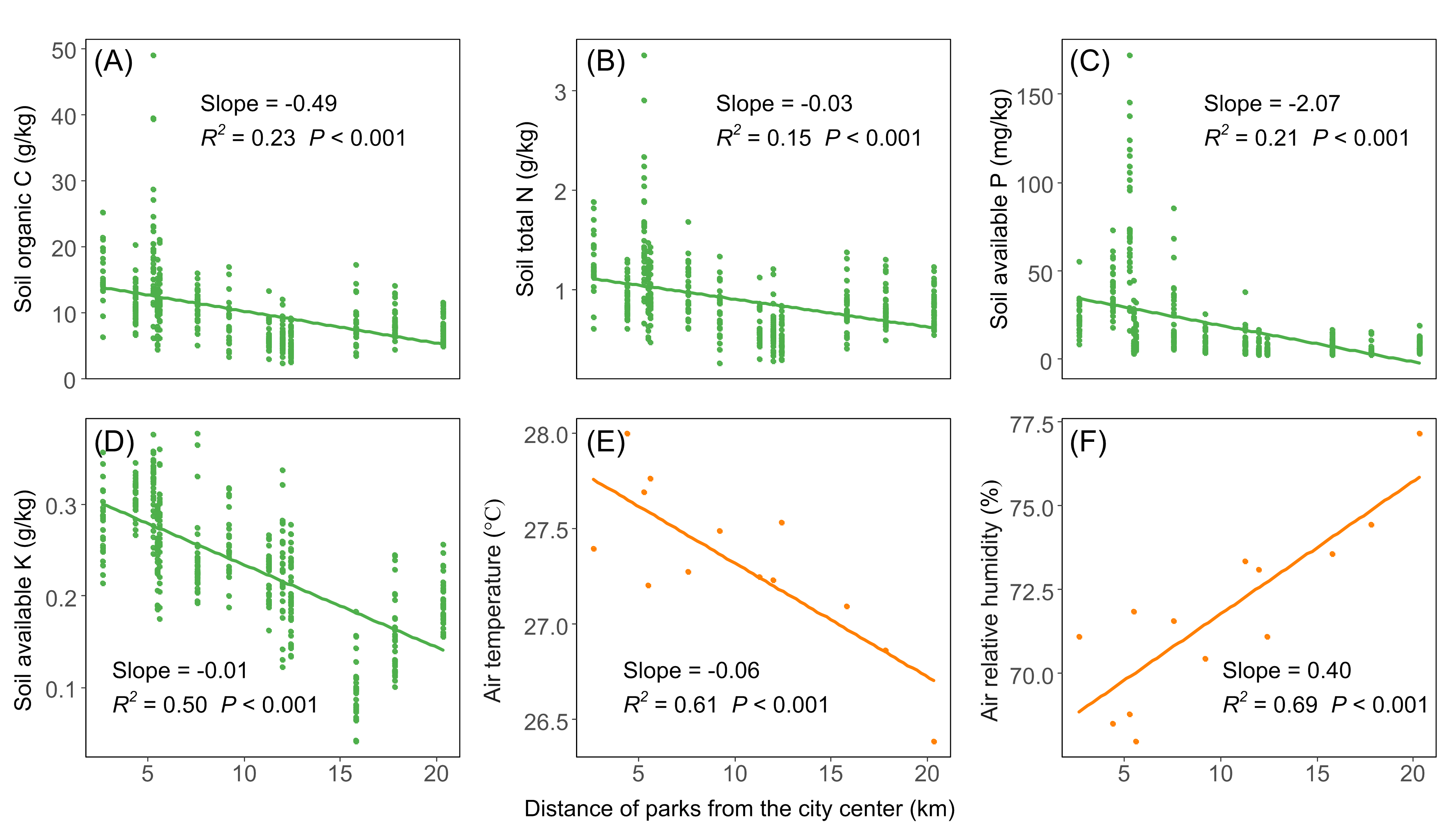

Supplement: Supplementary Figure 4 — Changes in (A–D) soil nutrients and (E,F) micro-climate along the urban–rural gradient, fitted with general linear models, with environmental factor as the response variable and distance as the predictor variable. [file Image_4.TIF]
