# Supplementary material for: Leaf Morphological and Nutrient Traits of Common Woody Plants Change Along the Urban–Rural Gradient in Beijing, China
Source: Front Plant Sci. 2021 Aug 26;12:682274. doi: 10.3389/fpls.2021.682274 (PMC8427184; doi:10.3389/fpls.2021.682274)
Supplement: Supplementary Table 1 — Basic profiles of the public parks selected. [file Table_1.docx]

## Table S1 Basic profiles of the public parks selected.

| **Parks** | **Distance from the city center (km)** | **Elevation (m)** | **Native or alien soils** | **Profile of irrigation** | | **Profile of fertilization** | | **Profile of pesticide use** | | **Profile of pruning** |
| --- | --- | --- | --- | --- | --- | --- | --- | --- | --- | --- |
|  |  |  |  | **Frequency** | **Water** | **Frequency** | **Fertilizer** | **Frequency** | **Pesticide** |  |
| Jingshan Park | 2.65 | 51.8 | Native | Once every 2 to 3 days | Tap water or groundwater | Twice a year | Organic compound fertilizer | Almost everyday | Imidacloprid | No pruning for all trees and *Lonicera maackii* (Rupr.) Maxim; Once a year for other shrubs |
| Nanguan Park | 4.36 | 44.9 |  | Once every 2 days | Tap water |  |  | Once a week | Imidacloprid, pyrethroids, diflubenzuron |  |
| Yuetan Park | 5.28 | 56.1 |  | Once every 2 to 3 days |  |  |  | Twice a month | Imidacloprid, propargite |  |
| Ditan Park | 5.53 | 47.6 |  | Once every 3 to 4 days | Reclaimed water or groundwater |  |  | Depends on the occurrence of pests | Imidacloprid |  |
| Qingnianhu Park | 5.64 | 47.7 |  |  | Tap water |  |  |  | Imidacloprid, pyrethroids, pyridaben |  |
| Shuangxiu Park | 7.63 | 49.8 |  |  | Reclaimed water |  |  |  | Imidacloprid |  |
| Taiyanggong Park | 9.2 | 42.9 |  | Once every 3 to 5 days |  | Twice a year for shrubs, no fertilizer for trees |  |  |  |  |
| Huangcaowan Country Park | 11.32 | 40.5 |  |  | Tap water | Once a year | Organic compound fertilizer, urea |  | Imidacloprid, dimethoate |  |
| Olympic Forest Park | 12.01 | 45.5 |  | Once every 2 to 3 days | Reclaimed water | Twice a year |  |  | Imidacloprid, pyrethroids |  |
| Yangshan Park | 12.38 | 44.8 |  | Once every 3 to 5 days |  |  |  |  | Imidacloprid, dimethoate |  |
| Dongxiaokou Forest Park | 15.78 | 34.5 |  |  | Groundwater | Twice a year for shrubs, no fertilizer for trees | Organic compound fertilizer | Once a year | Imidacloprid |  |
| Taiping Country Park | 17.83 | 39.5 |  | Once every 3 to 4 days |  | Twice a year |  | Depends on the occurrence of pests | Imidacloprid, avermectin |  |
| Banta Country Park | 20.34 | 40.3 |  |  | Reclaimed water |  |  |  | Imidacloprid, phoxim |  |
