# Supplementary material for: Leaf Morphological and Nutrient Traits of Common Woody Plants Change Along the Urban–Rural Gradient in Beijing, China
Source: Front Plant Sci. 2021 Aug 26;12:682274. doi: 10.3389/fpls.2021.682274 (PMC8427184; doi:10.3389/fpls.2021.682274)
Supplement: Supplementary Table 2 — Plant height and diameter at breast height. [file Table_2.docx]

## Table S2 Plant height and diameter at breast height.

| Species | Plant height (m) | Diameter at breast height (cm) |
| --- | --- | --- |
| *Pinus tabuliformis* Carr. | 5.3 ± 1.4 | 19.0 ± 3.7 |
| *Acer truncatum* Bunge | 6.7 ± 1.4 | 22.4 ± 6.5 |
| *Salix babylonica* L. | 9.2 ± 2.8 | 31.6 ± 9.7 |
| *Sophora japonica* L. | 8.4 ± 2.1 | 28.3 ± 10.8 |
| *Fraxinus chinensis* Roxb. | 8.4 ± 3.4 | 27.0 ± 10.2 |
| *Ginkgo biloba* L. | 7.8 ± 1.9 | 22.4 ± 8.7 |
| *Forsythia suspensa* (Thunb.) Vahl | 2.0 ± 0.6 | NA |
| *Lonicera maackii* (Rupr.) Maxim. | 3.1 ± 0.5 | NA |
| *Lagerstroemia indica* L. | 2.0 ± 0.4 | NA |
| *Kerria japonica* (L.) DC. | 0.9 ± 0.3 | NA |
| *Jasminum nudiflorum* Lindl. | 1.0 ± 0.4 | NA |
